# *LINC00669* promotes lung adenocarcinoma growth by stimulating the Wnt/β‐catenin signaling pathway

**DOI:** 10.1002/cam4.5604

**Published:** 2023-01-09

**Authors:** Jinhong Zhu, Kui Cao, Ping Zhang, Jianqun Ma

**Affiliations:** ^1^ Department of Clinical Laboratory, Biobank Harbin Medical University Cancer Hospital Harbin China; ^2^ Department of Thoracic Surgery Harbin Medical University Cancer Hospital Harbin China

**Keywords:** *LINC00669*, LUAD, prognostic signature, tumor immune environment, Wnt

## Abstract

Lung cancer poses severe threats to human health. It is indispensable to discover more druggable molecular targets. We identified a novel dysregulated long non‐coding RNA (lncRNA), *LINC00669*, in lung adenocarcinoma (LUAD) by analyzing the TCGA and GEO databases. Pan‐cancer analysis indicated significantly upregulated *LINC00669* across 33 cancer types. GSEA revealed a tight association of *LINC00669* with the cell cycle. We next attempted to improve the prognostic accuracy of this lncRNA by establishing a risk signature in reliance on cell cycle genes associated with *LINC00669*. The resulting risk score combined with *LINC00669* and stage showed an AUC of 0.746. The risk score significantly stratified LUAD patients into low‐ and high‐risk subgroups, independently predicting prognosis. Its performance was verified by nomogram (*C*‐index = 0.736) and decision curve analysis. Gene set variation analysis disclosed the two groups' molecular characteristics. We also evaluated the tumor immune microenvironment by dissecting 28 infiltrated immune cells, 47 immune checkpoint gene expressions, and immunophenoscore within the two subgroups. Furthermore, the risk signature could predict sensitivity to immune checkpoint inhibitors and other anticancer therapies. Eventually, in vitro and in vivo experiments were conducted to validate *LINC00669'*s function using qRT‐PCR, CCK8, flow cytometry, western blot, and immunofluorescence staining. The gain‐ and loss‐of‐function study substantiated *LINC00669'*s oncogenic effects, which stimulated non‐small cell lung cancer cell proliferation but reduced apoptosis via activating the Wnt/β‐catenin pathway. Its oncogenic potentials were validated in the xenograft mouse model. Overall, we identified a novel oncogenic large intergenic non‐coding RNA (lincRNA), *LINC00669*. The resulting signature may facilitate predicting prognosis and therapy responses in LUAD.

## Graphical abstract

We identified and validated a novel oncogenic lincRNA of LINC00669. The resulting signature based on LINC00669‐assoicated cell cycle genes may facilitate predicting prognosis and therapy responses in LUAD.

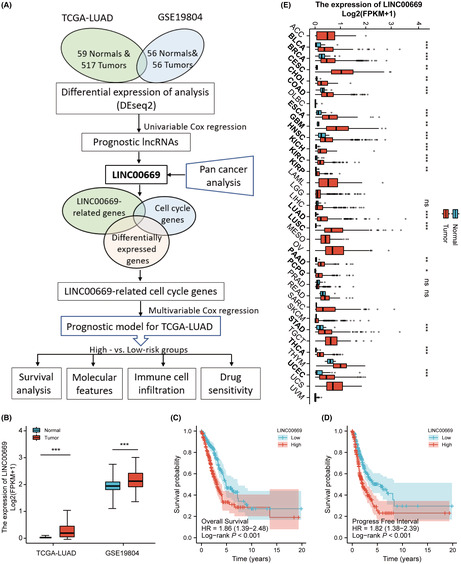

## 1 INTRODUCTION

Cancer is primarily a disease of aging. The incidence and associated death exponentially increase for most types of cancer among populations ≥65 years old, which is partially attributed to growing somatic mutations with aging.^1^ It is evaluated that mutations approximately rise by 0.077 per megabase per year.^2^ Lung cancer, the deadliest cancer in the world, was evaluated with an annual 2.20 million new cases and 1.79 million deaths.^3^, ^4^ The non‐small cell lung cancer (NSCLC) is the primary subtype of lung cancer. Dramatic progress has been made in treating NSCLC, including novel targeted therapies with enhanced efficacy, lessened toxicity, and immune‐checkpoint inhibitors. Unfortunately, most advanced NSCLCs, initially respond to current treatments, often develop resistance, and ultimately progress.^5^, ^6^ Thus, it is indispensable to deepen the understanding of molecular and cellular mechanisms underpinning lung tumorigenesis to develop new therapeutic strategies and rational combination therapies to overcome resistance.

Long non‐coding RNAs (lncRNAs) are an essential class of ubiquitously expressed genes regulating various biological functions,^7^, ^8^ which are RNAs containing more than 200 nucleotides without protein‐coding potential. Based on genomic locations relative to near protein‐coding genes, lncRNAs are classified into sense or antisense lncRNAs overlapping with host genes, intronic lncRNAs, enhancer lncRNA transcribed from enhancer regions, divergent lncRNAs, and large intergenic non‐coding RNAs (lincRNAs) originating from intergenic regions of genomes.^9^ Substantial evidence implicates lncRNAs in lung carcinogenesis, many of which might be utilized as biomarkers assisting risk prediction, diagnosis, and prognosis or therapeutically targeted in lung cancer.^10^, ^11^, ^12^, ^13^ *LncRNA MARCKSL1‐2* was remarkably downregulated in docetaxel (DTX)‐resistant lung adenocarcinoma cells, while overexpression of this lncRNA in tumor cells reversed DTX resistance.^14^ Hao and colleagues found that five lncRNAs (*SCAMP1‐AS1*, *LINC00892*, *LINC01481*, and *AC004812.2*, *AC099522.2*) could be potentially used as diagnostic biological markers for screening N2 metastasis in pT1 lung adenocarcinoma (LUAD).^15^ Elevated expression levels of lncRNA *LCIIAR* foreseen a poor prognosis in lung cancer.^16^ Tumor‐suppressing lncRNAs are also reported.^14^, ^17^ However, the field of lncRNAs remains to be intensively explored. It is urgent to discover novel lncRNAs and delineate their regulation and functional significance concerning lung cancer. By analyzing differentially expressed lncRNAs between normal and cancerous lung tissues in The Cancer Gene Atlas (TCGA) project and GSE19804, we found an intergenic lncRNA, *LINC00669*, is overexpressed and associated with unfavorable prognosis in LUAD. Only one research with experimental data on *LINC00669* has been published, indicating that *LINC00669* accelerated nasopharyngeal cancer cell proliferation and invasion through sequestering the JAK/STAT suppressor SOCS1.^18^

The aberrant activation of the wingless related‐integration site (Wnt)/β‐catenin signaling pathway contributes to tumor progression and drug resistance in NSCLC.^19^, ^20^, ^21^ In the absence of proper external stimuli, cytoplasmic β‐catenin is subjected to β‐TrCP‐dependent proteasomal degradation, with the assistance of a multi‐protein complex involving glycogen synthase kinase‐3α (GSK3α), GSK3β, adenomatous polyposis coli (APC), Axin, casein kinase I (CKI), and Disheveled. Once the ligands activate the Wnt signaling, the destruction protein complex is disintegrated, and β‐catenin is stabilized and translocated into nuclei. In the nucleus, β‐catenin binds to transcription factors T‐cell factor/Lymphoid enhancer factor 1 (TCF/LEF 1), thereby facilitating the transcription of target genes (e.g., *c‐MYC* and *CYCLIN D1*).

Here, our bioinformatics analyses showed that *LINC00669* is strongly associated cell cycle. The signature out of *LINC00669‐*associated cell cycle genes robustly predicted patient prognosis, intratumoral immune cell infiltration, and immunotherapy response in LUAD. Furthermore, experimental evidence indicated that *LINC00669* promoted lung cancer progression through the Wnt signaling pathway.

## 2 MATERIALS AND METHODS

### 2.1 Bioinformatics

#### 2.1.1 Retrieval of differentially expressed lncRNAs for LUAD

The RNA‐Seq dataset of the lung adenocarcinoma cohort was downloaded from The Cancer Genome Atlas (TCGA) (https://nci.nih.gov/tcga/), along with clinical information. We first pinpointed 282 dysregulated lncRNAs between 517 tumors and 59 normal tissues (log2FC >1 or < −1, adjusted *p* < 0.05), which was validated with a GSE19804 LUAD cohort. This dataset contained 60 pairs of lung tumors and adjacent normal tissues, among which RNA‐seq data from 56 LUAD tumors and matched adjacent normal lung tissue specimens were used for this study.^22^ After excluding samples without clinical and survival information, 506 tumor samples in the TCGA‐LUAD cohort remained for the following analysis. Totally 113 of 282 dysregulated lncRNA were confirmed in the GSE19804 dataset. Univariate Cox regression analysis was performed to screen the 113 lncRNAs for prognostic significance against overall survival (OS) in the TCGA‐LUAD cohort. The data were processed as reported before.^17^, ^23^, ^24^, ^25^ Eventually, *LINC00669*, associated with survival, was chosen for the study. Moreover, the TCGA database enabled us to evaluate the expression levels of *LINC00669* across 33 types of cancers.

#### 2.1.2 Gene set enrichment analysis

Gene set enrichment analysis (GSEA) was conducted to explore the function of *LINC00669* using GSEA software 4.0.0. Other than the TCGA database, we accessed the Cancer Cell Line Encyclopedia (CCLE) database (https://sites.broadinstitute.org/ccle) to acquire the RNA‐seq data of 119 NSCLC cell lines. The median expression level of *LINC00669* was used to divide NSCLC cell lines into *LINC00669* ^low^ and *LINC00669* ^high^ subsets. All analyses were performed with the TCGA‐LUAD cohort thereafter without notification.

#### 2.1.3 A risk signature of LINC00669‐associated cell cycle genes

With Pearson's correlation analysis, 15,868 *LINC00669*‐correlated genes were obtained from the TCGA‐LUAD cohort (*p* < 0.05, |correlation coefficient| ≥ 0.1). We also secured 14,162 differentially expressed genes in LUAD (*p* < 0.05, |log2FoldChange| >1). Besides, 1056 cell cycle genes were retrieved from the CancerSEA database (http://biocc.hrbmu.edu.cn/CancerSEA/). A Venn diagram approach was used to select *LINC00669*‐related cell cycle genes that are differentially expressed in LUAD. The univariate Cox regression analysis further filters out genes without significant association with OS. A prognostic signature was constructed with the remaining significant genes using the multivariable Cox regression model. After generating the signature, the expression levels of genes of interest and risk coefficients were integrated to appoint each patient a risk score as published previously.^17^, ^23^, ^24^, ^25^ Using the R package survivalROC, the time‐dependent receiver operating characteristic (ROC) curves were generated, with the area under the ROC curve (AUC) as a measurement of the prognostic signature predictive accuracy. The maximum Youden's *J* statistic (*J* = sensitivity + specificity −1) was determined for the time‐dependent ROC curve with the biggest AUC and used as a cutoff to define patients with different risks. A Kaplan–Meier survival analysis was carried out with the R package rms, followed by uni‐ and multivariable Cox regression analyses.

#### 2.1.4 Evaluation of the risk signature by a Nomogram

Next, a Cox‐based nomogram was generated, coupled with calibration curves. We also performed decision curve analysis (DCA) to quantify the net benefit for the nomogram. This analysis assisted the understanding of the clinical benefit of the risk model.

#### 2.1.5 Gene set variation analysis for molecular characteristics

Gene set variation analysis (GSVA) was adopted to interrogate the differences between the two subgroups concerning molecular features. The relevant gene sets were down from HALLMARK and Kyoto Encyclopedia Genes and Genomes (KEGG) databases, and the R package of GSVA was chosen for the analysis.

### 2.2 Evaluation of tumor immune microenvironment

Intratumoral infiltrating immune cells account for a crucial component of the tumor microenvironment (TME).^26^ The metagene signatures recapitulating 28 intratumoral immune cell subpopulations have been developed for Pan‐cancer before.^27^ By adopting the metagene dataset, the R package “GSVA” was applied to execute the single‐sample GSEA (ssGSEA) algorithm to assess fractions of 28 immune cell subsets infiltrated in tumor samples. Immune checkpoints (ICPs) are also believed to contribute to tumor immunogenicity.^28^ The expression levels of 47 ICP genes were determined for the two subgroups of the TCGA‐LUAD cohort.

In addition, Charoentong and colleagues developed The Cancer Immunome Atlas (https://tcia.at/) in 2017 by integratively analyzing the immune landscapes and antigenomes across 20 solid tumors.^27^ They also quantitatively evaluated tumor immunogenicity with an immunophenoscore (IPS), with scores of 0 and 10 depicting the minimal and maximal tumor immunogenicity, respectively.^27^ ISP was calculated for LUAD patients in the two risk groups.

#### 2.2.1 Predicting the patients' response to anticancer therapy

IPS has been shown to perform robustly in predicting the patients' sensitivity to ICI treatment.^27^ Here, we acquired IPS data to interrogate the response to ICIs between the two risk groups.

Moreover, we checked the signature's capacity to predict other clinical drug responses in LUAD patients. The pRRophetic is an R package able to predict the sensitivity of 138 drugs from tumor gene expression profiles.^29^, ^30^ We estimated the log (half inhibitory concentration IC50) of anticancer drugs in the LUAD patients via this algorithm. Wilcoxon signed‐rank test was executed to compare the log (IC50) between the two groups.

### 2.3 Experimental validations

#### 2.3.1 Cell culture and establishment of stable cell lines

We purchased human bronchial epithelial (HBE) cells and NSCLC cell lines, H1975, H1299, H358, and A549, from ATCC. HBE cells were maintained in Dulbecco's Modified Eagle's Medium (DMEM) with the addition of 10% fetal bovine serum (Sigma) at 37°C and 5% CO_2_, whereas cancer cell lines were cultured in RPMI‐1640 medium. Lentiviral vectors (LV) were used to achieve stable expression or knockdown of *LINC00669* (NR_024391.1) in target cells.

Lentiviral vectors (pLVX‐Puro) carrying human *LINC00669* cDNA sequence were constructed as described previously.^17^ The pLKO.1‐puro derived from HIV‐1 was used as a transferring and cloning vector of shRNA‐*LINC00669*. The sequences of shRNAs targeting *LINC00669* were shRNA‐1: 5′‐TTTAGATTAAGCACTATCC‐3′, shRNA‐2: 5′‐TTGATGTTGAAGAACTTTG‐3′, shRNA‐3: 5′‐TAATTTACTAGAGCTTACC‐3′. H1299 cells were infected with empty vectors or lentiviruses overexpressing *LINC00669*. In parallel, H358 cells were used for silencing *LINC00669* by LV‐sh shRNAs. Cells infected by lentiviruses underwent selection with puromycin to establish stable cell lines.

#### 2.3.2 RNA isolation and quantitative RT‐PCR

After discarding the culture medium, we harvested NSCLC cells, and total RNAs were isolated using the Trizol‐based protocol (Thermo Fisher Scientific). The RNA concentration of each sample was measured with a Nanodrop1000 spectrophotometer (NanoDrop). Complementary DNAs (cDNAs) were produced from 1 μg of RNA template using the Thermo Scientific™ RevertAid™ First Strand cDNA synthesis kit. The SYBRGreen mix (Thermo Fisher Scientific) was employed to accomplish quantitative PCR (qPCR) on an ABI Applied Biosystems 7300 platform. Amplification reaction yielded a PCR product of 271 bps for *LINC00669* (Forward primer: 5′ GACATCAGCATCTCAGTTCTC 3″ and Reverse primer: 5′ T'GGTCAACCTCTTCTAATCC 3″). Data were analyzed with ABI Prism 7300 SDS software, using The 2^−ΔΔCt^ method to assess relative gene expression. Expression analyses of *LINC00669* were normalized to the housekeeping gene (GAPDH: Forward Primer: 5′ AATCCCATCACCATCTTC 3′ and Reverse primer: 5’ A'GCTGTTGTCATACTTC 3′).

#### 2.3.3 CCK8 assay

We used a cell counting kit‐8 kit (CCK8; Signalway antibody) to determine whether the gain or loss of function of *LINC00669* affects NSCLC cell proliferation. Cell suspensions were added to a 96‐well tissue culture plate (3 × 10^3^ cells per well) and then grew in a 5% CO_2_ humidified incubator at 37°C. The assay was performed in triplicate. After incubation for 24, 48, and 72 h, the medium was removed, and the CCK8 assay was conducted following the manufacturer's direction. Finally, cell proliferation was presented as optical density (OD) measured by an ELISA plate reader at the wavelength of 450 nm.

#### 2.3.4 Analysis of cell apoptosis

NSCLC cells were stained with the Annexin V‐FITC/PI Apoptosis Detection Kit (Beyontime Biotechnology). Cells were counted on a CytoFLEX Flow Cytometer (Becton Dickinson). In the case of investigating whether *LINC00669* stimulates NSCLC cell proliferation by upregulating the Wnt/β‐catenin pathway. H1299 cells stably overexpressing *LINC00669* were treated with XAV939 (MedChemExpress LLC), an inhibitor of the Wnt/β‐catenin pathway.

#### 2.3.5 Western blot

NSCLC cells were harvested and lysed according to routine laboratory protocol. After using the BCA protein assay kit (Thermo Fisher Scientific) to determine total protein concentrations, an SDS‐PAGE was run to separate the identical amounts of total proteins, which were next transferred onto a nitrocellulose membrane. The blots were preincubated with 5% nonfat dry milk and reacted with the indicated primary antibody. The primary antibodies used in this study were as follows: antibodies against PCNA, Ki67, Survivin, Cleaved Caspase 3 (Abcam), H3 (Proteintech), GAPDH (Proteintech), β‐catenin (Cell signaling Technology). Secondary HRP‐conjugated antibodies (SolelyBio), detecting mouse‐ or rabbit‐derived primary antibodies, were used when appropriate. We finally visualized blots with Enhanced Chemiluminescence (Thermo Fisher Scientific).

#### 2.3.6 Turmirgenicity assay in mice

The animal protocol was approved by the Institutional Review Board of Harbin Medical University Cancer Hospital. Female athymic nude mice at 5–6 weeks of age were used in this study (SLAC Laboratory Animal Co., Ltd). We kept mice under specific pathogen‐free conditions. Mice were randomly assigned into control or experimental groups (*n* = 6 for shCtrl vs. sh*LINC00669*). H358 cells with stable knockdown of LINC00669 or control cells (2 × 10^6^) suspended in PBS were subcutaneously implanted into the left flanks of mice. Tumor length and width were measured every 3 days using digital calipers (ProSciTech Pty Ltd.). Mice were sacrificed on the 33 days from the beginning of cell injection, and tumors were collected and weighed. Tumor specimens were sequentially fixed in formalin and embedded in paraffin, followed by histological analysis. Immunofluorescence (IF) staining was performed on tumor sections to determine the expression levels of Ki67 and proliferating cell nuclear antigen (PCNA). Primary antibodies for Ki67 (Abcam), PNCA (Proteintech), and Alexa Fluor 488‐labeled anti‐rabbit IgG (H + L) (Beyotime Biotechnology) were used for IF staining.

#### 2.3.7 Statistical analysis

We conducted all statistical analyses using the SPSS version 22 (SPSS, Inc.). The Student's *t*‐test was conducted to check the significant differences between the two groups. A one‐way analysis of variance (ANOVA) was employed to compare the differences among groups ≥3. The significant results were subjected to post hoc tests. Moreover, a two‐way ANOVA was applied as needed. All analyses were two‐sided. The nominal 0.05 significance level was adopted throughout the study.

## 3 RESULTS

### 3.1 Identification of an oncogenic lncRNA‐*LINC00669* in lung adenocarcinoma

**FIGURE 1:**
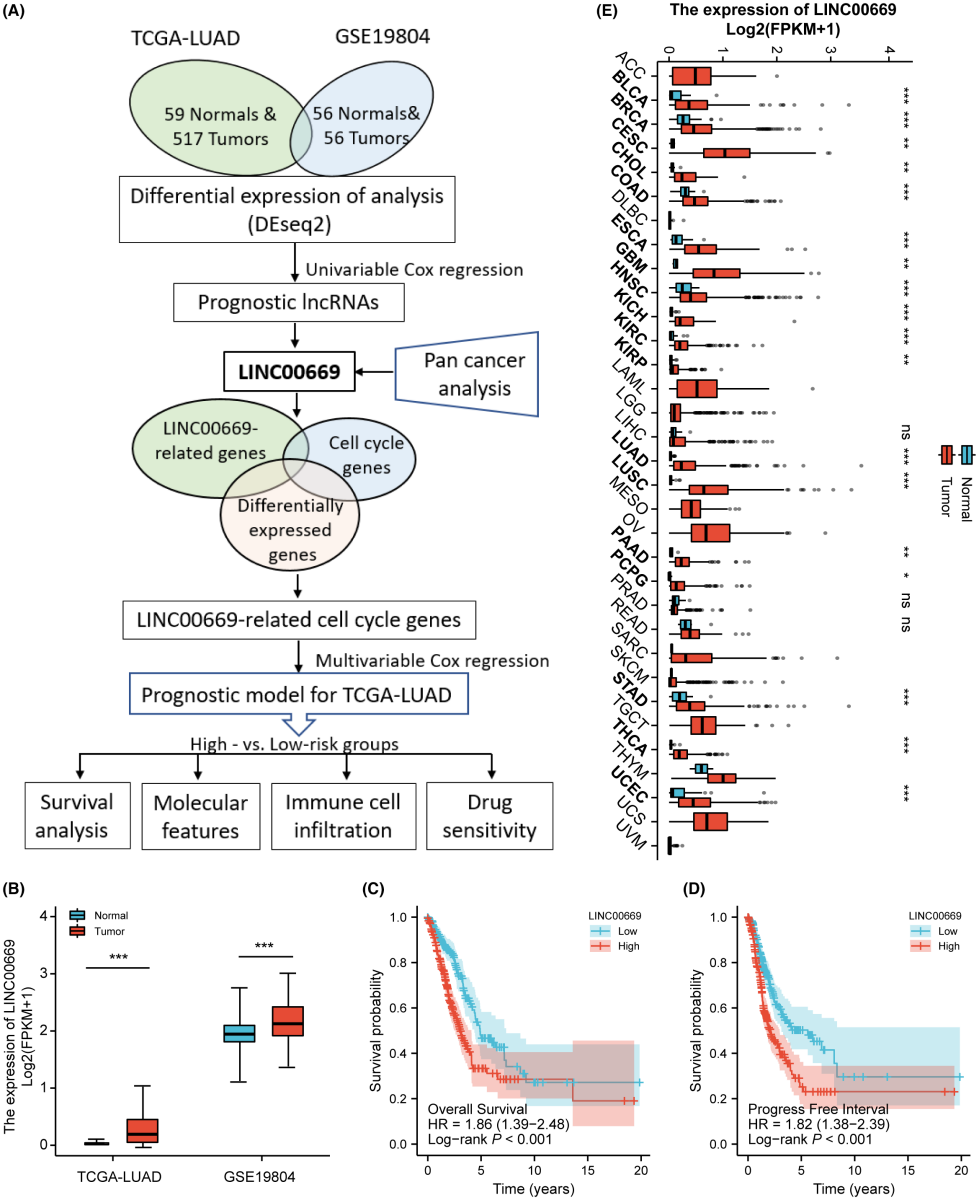
Identifying a novel oncogenic lincRNA in lung adenocarcinoma (LUAD). (A) The flow chart of bioinformatic analysis. (B) *LINC00669* was significantly upregulated in the TCGA and GSE19804 LUAD cohorts. (C, D) *LINC00669* was associated with overall survival (OS) and progression‐free interval (PFI) in the TCGA‐LUAD cohort. (E) Pan‐cancer analysis showed upregulation of *LINC00669* in various types of cancer *p < 0.05, **p < 0.01, ***p < 0.001.

The study flow is shown in Figure 1A. We identify 282 lncRNAs and 1960 genes differentially expressed in the TCGA and GSE18804 LUAD cohorts, respectively (Figure S1). Venn diagram showed that 113 lncRNAs were dysregulated in both cohorts (Figure S1). The prognostic values of lncRNAs were calculated using univariate Cox regression analysis. Among the prognostic lncRNAs (Table S1), *LINC00669*, an intergenic lncRNA, was upregulated in both LUAD cohorts and associated with OS and progression‐free interval (PFI) (Figure 1B–D). Pan‐cancer analysis elucidated that *LINC00669* was significantly augmented in 18 out of 33 types of cancer (Figure 1E). These results suggest that *LINC00669* is potentially a crucial oncogenic lincRNA.

### 3.2 Construction of a prognostic signature of *LINC00669* ‐associated cell cycle genes in LUAD

**FIGURE 2:**
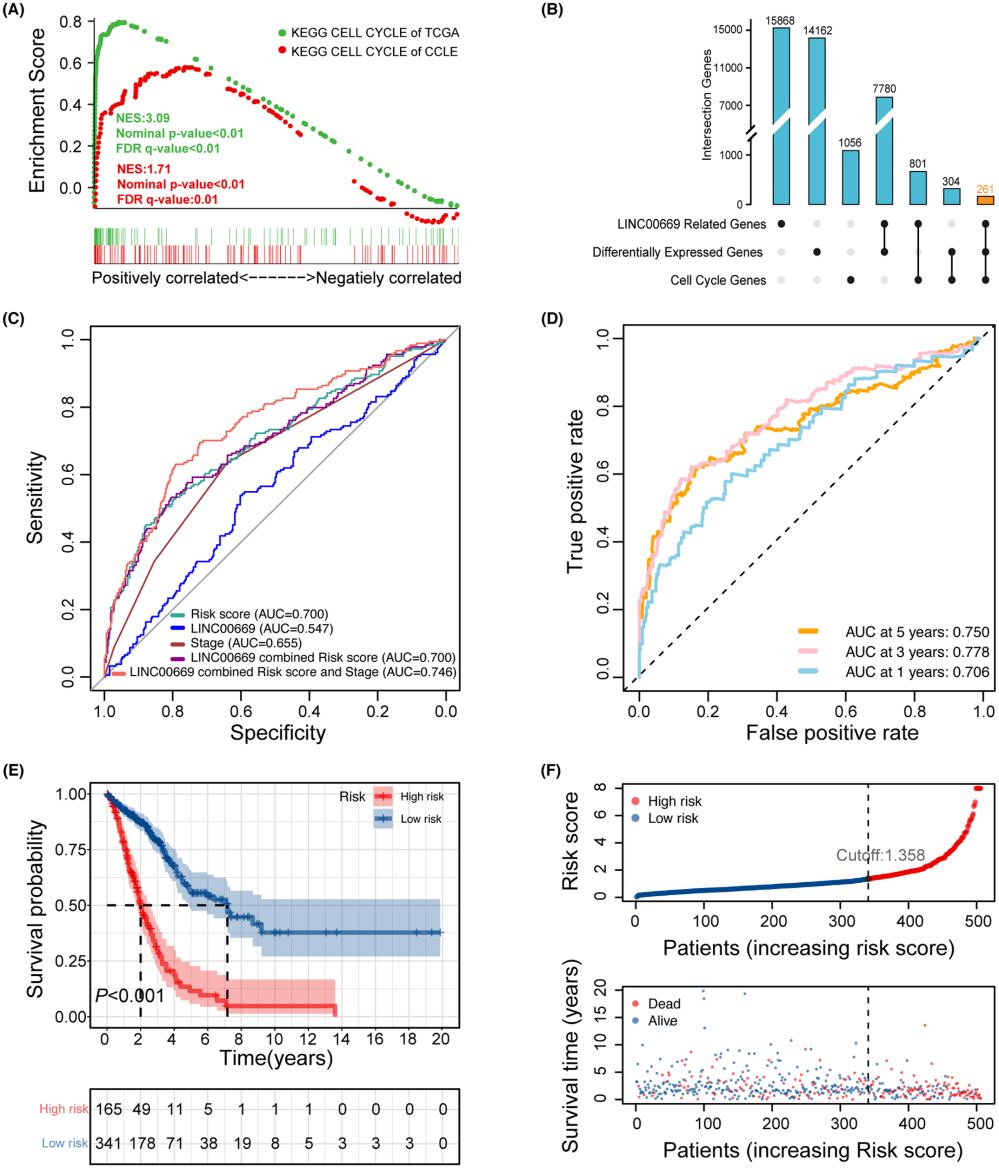
Development and evaluation of a signature with *LINC00669*‐related cell cycle genes. (A) Gene Set Enrichment Analysis (GSEA) on the TCGA and CCLE datasets suggests the association between *LINC00669* and cell cycle in lung cancer. (B) Schematic diagram of overlapped genes among *LINC00669*‐related genes, differentially expressed genes in the TCGA‐LUAD cohort, and cell cycle genes from the CancerSEA database. A multivariate Cox regression was used to establish a signature with *LINC00669*‐related cell cycle genes. (C) Receiver operator characteristic (ROC) curves were plotted for the risk score, *LINC00669*, stage, and combined variables regarding OS. (D) Time‐dependent ROC curves for the risk score in the TCGA‐LUAD cohort. (E) Kaplan–Meier survival curves of OS time for the low ‐ and high‐risk patients defined by the risk score. (F) The risk score distribution and corresponding survival status of the TCGA‐LUAD cohort.

Because of *LINC00669*'s oncogenic potential, we interrogated its functional relevance in LUAD by performing GSEA with RNA‐seq data of the TCGA‐LUAD cohort and 119 NSCLC cell lines from the CCLE database. GSEA revealed that *LINC00669‐*correlated genes were significantly associated with the cell cycle pathway in both datasets (Figure 2A), suggesting the vital role of this lncRNA in cell cycle regulation. And then, we extracted 261 genes shared by three gene lists (*LINC00669‐*correlated genes, differentially expressed genes, and cell cycle genes), which were termed *LINC00669*‐associated cell cycle genes (Figure 2B). Among them, 82 genes showed prognostic significance. A multivariate Cox regression analysis was adopted to generate a risk signature out of *LINC00669*‐associated cell cycle genes by eliminating genes not independently predicting prognosis for the TCGA‐LUAD patients. The prognostic signature involved 25 genes (Table 1), based on which each patient received a risk score. The ROC curves were plotted to evaluate the predicting accuracy of the prognostic signature. The AUC for the risk score derived from the prognostic signature alone was 0.700, which outperformed *LINC00669* or stage alone in distinguishing favorable or poor clinical outcomes in LUAD patients (Figure 2C). While in combination with *LINC00669* and stage, the prognostic accuracy of the risk signature was further advanced (AUC = 0.746) (Figure 2C). The time‐dependent AUCs were drawn for the risk score, illustrating the highest AUC of 0.778 at 3 years (Figure 2D). Using the 3‐year ROC, we determined the best cutoff value of 1.358 for the risk score.

**TABLE 1:** Coefficients of genes in the risk signature

Gene	Coefficients	Hazard ratio	*p* value
ANLN	0.616986	1.853334	8.48 E‐05
ARHGAP11A	0.792623	2.209183	0.002382
CCNB1	−0.36721	0.692667	0.101634
CCNB2	−0.50822	0.601564	0.10471
CDC25C	0.583821	1.792875	0.021893
CDC6	−0.83583	0.433512	0.000266
DEPDC1	−0.52999	0.588611	0.056347
DEPDC1B	0.522867	1.686856	0.026503
ECT2	0.28374	1.328087	0.067462
KIF23	−0.57792	0.561065	0.099807
KPNA2	0.502051	1.652106	0.030071
LDHA	0.289649	1.335958	0.04077
LINC01537	1.927698	6.873671	7.39 E‐08
MAD2L1	−0.62434	0.535616	0.009288
NCAPG	−0.53981	0.58286	0.061456
NEIL3	0.269774	1.309669	0.025264
NUSAP1	−0.71988	0.486813	0.009199
PAICS	0.314302	1.369304	0.082648
PCLAF	0.620302	1.859489	0.004172
PLEK2	0.133664	1.143009	0.154536
PLK1	0.708677	2.031302	0.001275
PRR11	−0.37234	0.689118	0.148668
RRM2	0.518073	1.67879	0.009877
TYMS	0.475823	1.609338	0.002626
UHRF1	−0.37162	0.689615	0.062229

### 3.3 Prognostic value of the risk signature

**FIGURE 3:**
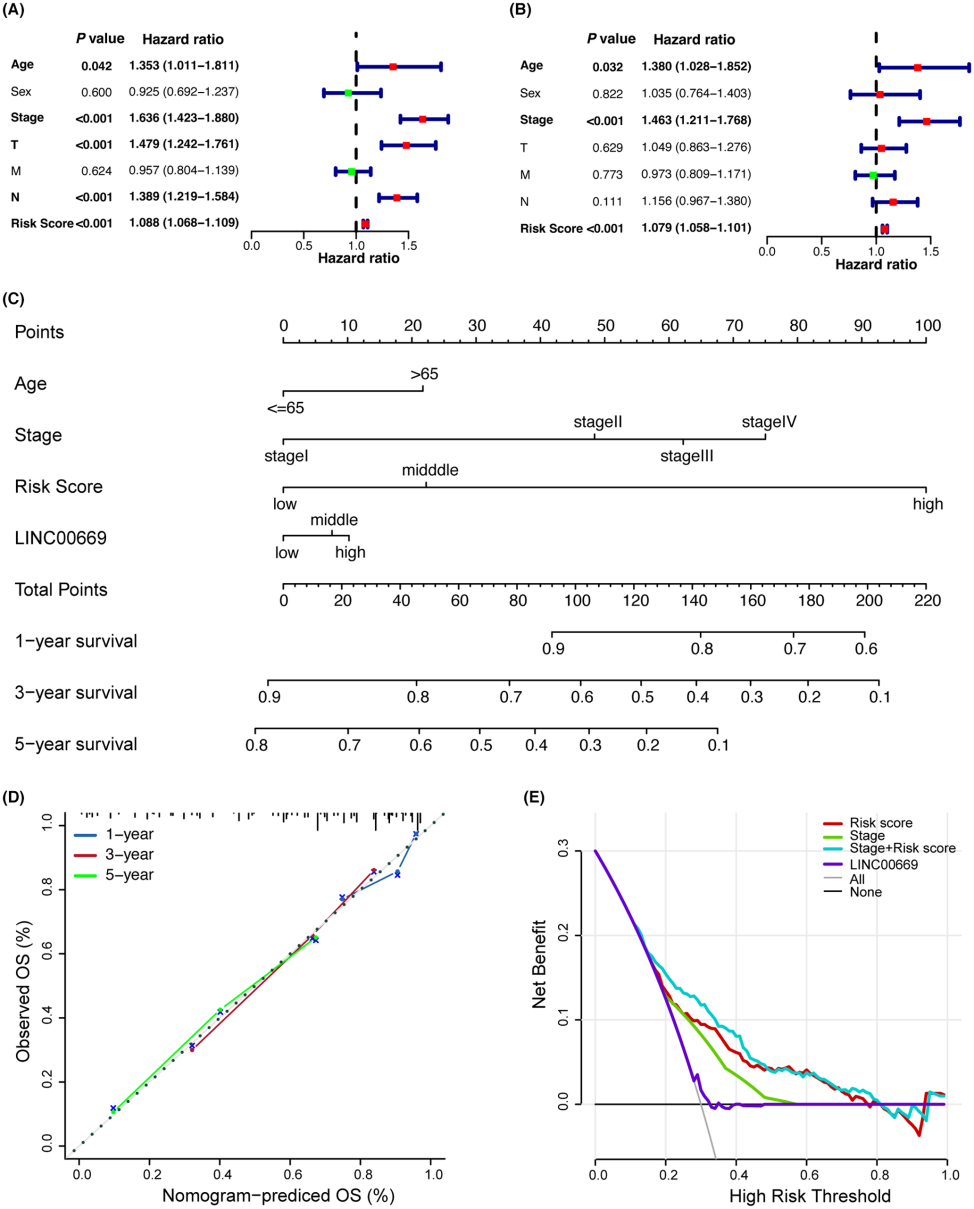
Assessment of the prognostic performance of risk score. (A, B) Univariate (left) and multivariate (right) Cox regression analysis. (C) Nomogram used to predict 3‐ and 5‐year survival. (D) 1‐, 3‐ and 5‐year calibration curves. (E) Decision curve analysis was performed to evaluate the net benefits of indicated variables. The black and gray lines represent a ‘predicting none’ and a ‘predicting all’ strategy, respectively.

This cutoff value was used to stratify patients into high‐risk and low‐risk groups. Kaplan–Meier survival analysis showed that the patients with low risk scores survived significantly longer than those with high risk scores (Figure 2E,F). Univariate Cox regression analysis was conducted to estimate the hazard ratio (HR) and 95% confidence interval (CI) of the risk score and other indicated variables, followed by a multivariate Cox regression analysis. As shown in Figures 3A,B, the risk score could independently predict prognosis in LUAD. Furthermore, we build a nomogram by integrating age, stage, risk score, and *LINC00669* (Figure 3C), accompanied by calibration curves (Figure 3D). The *C*‐index of the nomogram was 0.736, suggesting a robust discriminating accuracy of the risk model. Decision curve analysis was employed to evaluate the performance of the nomogram in evaluating the prognosis of LUAD patients. The risk score combined with the stage favored the best decision‐making, as revealed by the net benefits (Figure 3E).

### 3.4 Differences in molecular characteristics between two groups

**FIGURE 4:**
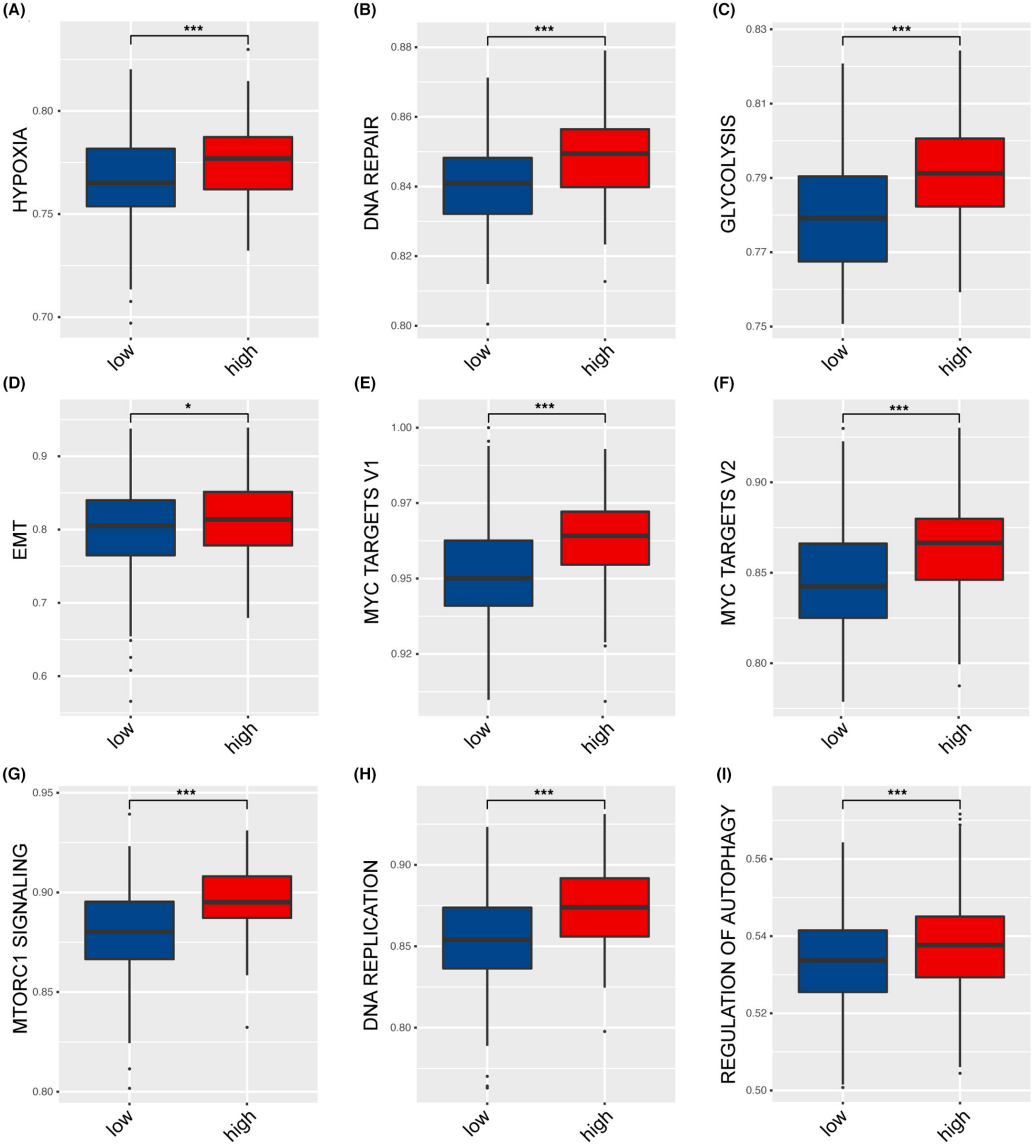
Gene set variation analysis (GSVA) to identify key molecular features for high‐ and low‐risk groups. (a–i) The following cellular events and signaling pathways significantly differ between the two groups: hypoxia (A), DNA repair (B), glycolysis (C), EMT (D), MYC targets v1 (E), MYC targets v2 (F), mTORC1 signaling (G), DNA replication (H), and regulation of autophagy (I).

We next interrogated whether some distinct molecular characteristics contribute to enhanced tumor malignancy in the high‐risk group compared with the low‐risk group. GSVA showed enhanced activity of some malignancy‐correlated pathways, including hypoxia, DNA repair, glycolysis, EMT, MYC targets V1, MYC targets V2, MTORC1 signaling, DNA replication, and regulation of autophagy (Figure 4A–I).

### 3.5 Estimation of tumor immune microenvironment

**FIGURE 5:**
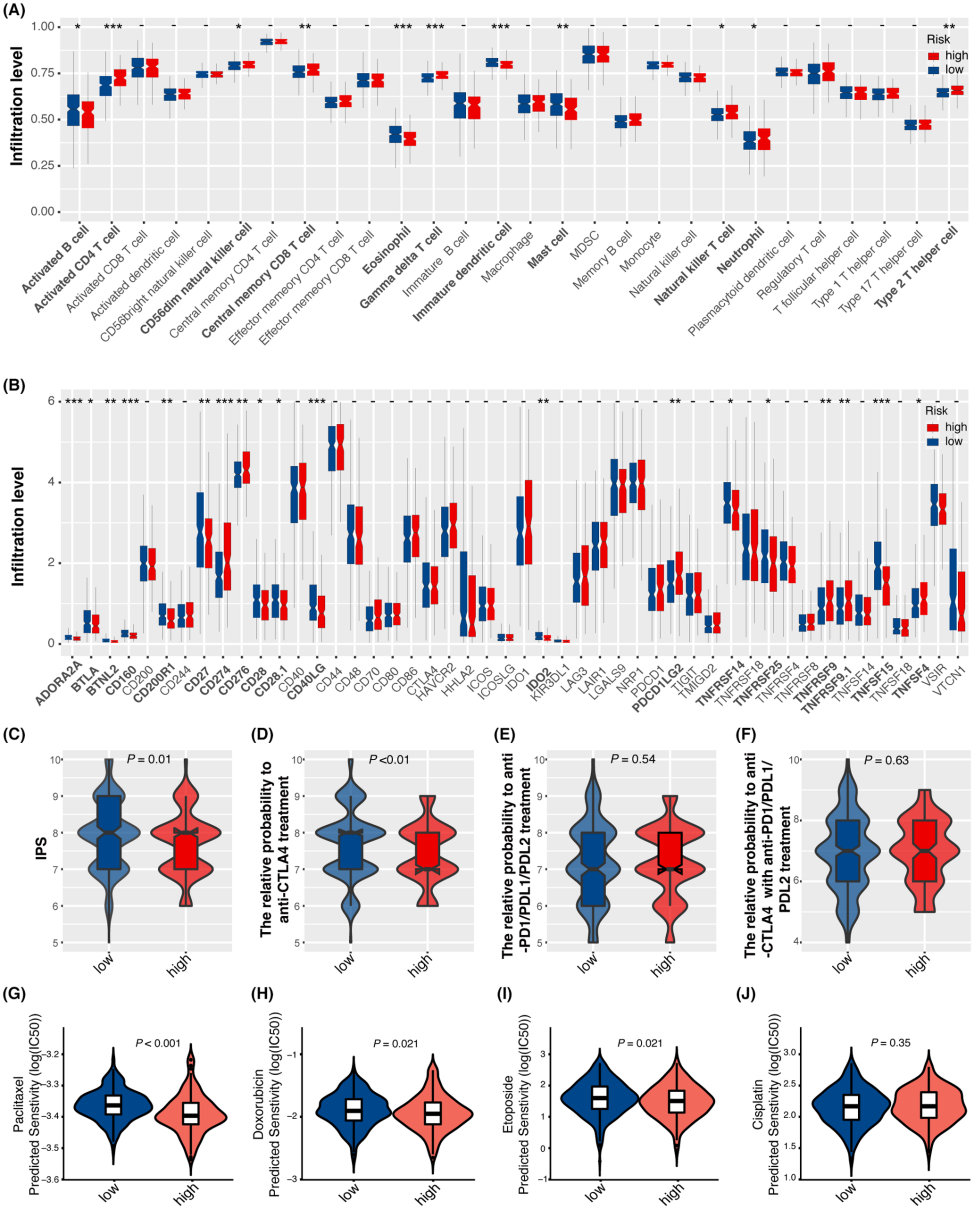
Comparison of tumor immune microenvironment and drug sensitivity between low‐ and high‐risk groups. (A) The composition of 28 immune cell subsets infiltrated in tumors. (B) Expression profiles of 47 immune checkpoint genes. (C) Immunophenoscores of low‐ and high‐risk groups. (D–F) Evaluation of response to anti‐PD1/PDL1/PDL2 (D), anti‐CTL4 (E), and combined therapies (F) using IPS approaches. (G–J) Predicted response to drugs, including paclitaxel (G), doxorubicin (H), etoposide (I), and cisplatin (J) using a pRRophetic algorithm.

Continuously interplaying between tumor cells and the host tumor immune microenvironment (TIME) may largely determine tumor prognosis and therapeutic efficacy.^26^ Due to the importance of TIME in tumor initiation, development, and drug response, we attempted to dissect tumor‐infiltrating immune cells in the two risk groups. Based on previously published signature genes for 28 immune cell subsets, we utilized ssGSEA to determine the fraction of immune cell infiltrates for single tumor samples in the TCGA‐LUAD cohort. Significant differences in tumor immune cell infiltration were found between the two risk groups, including activated B cells, activated CD4+ T cells, CD56^dim^ nature killer cells, central memory CD8+ T cells, gamma delta T cells, immature dendritic cells, mast cells, natural killer T cells, neutrophil, and type 2 T helper cells (Figure 5A). Moreover, ICPs also play essential roles in tumor immunogenicity. By comparing the expression of 47 ICPs between the two groups, we found that 19 of them were differentially expressed between the two groups, such as *CD160*, *BTLA*, *BTNL2*, *CD200R1*, *CD27*, *CD274*, *CD28.1*, *CD276*, *CD28*, *CD40LG*, *IDO2*, *ADORA2A*, *PDCD1LG2*, *TNFRSF14*, *TNFRSF25*, *TNFRSF9*, *TNFRSF9.1*, *TNFSF15*, and *TNFSF4* (Figure 5B). IPS, the most comprehensive immune determinant, is positively correlated with tumor immunogenicity. Our results indicated that the low‐risk group exhibited significantly higher IPS than the high‐risk group, suggesting enhanced tumor immunogenicity in the group with low risk (Figure 5C).

### 3.6 Prediction of patient response to anticancer treatments

A previous study indicated that IPS is an effective predictor of response to ICP blockade.^27^ By applying this approach to the TCGA‐LUAD cohort, we demonstrated that the low‐risk group tended to show a response to CTLA4 blockers compared with the high‐risk group (Figure 5D). However, the two groups displayed no significant differences regarding the responses to anti‐PD1/PDL1/PDL2 treatment or the combined therapy (Figure 5E,F).

We also investigated the risk score‘s ability to predict response to commonly used drugs for NSCLC. By analyzing RNA‐seq data with the pRRophetic algorithm, the resulting log (IC50) unveiled that the high‐risk group was significantly more sensitive to paclitaxel, doxorubicin, and etoposide than the low‐risk group (Figure 5G–J).

### 3.7 Impacts of *LINC00669* on apoptosis and proliferation of lung cancer cells

**FIGURE 6:**
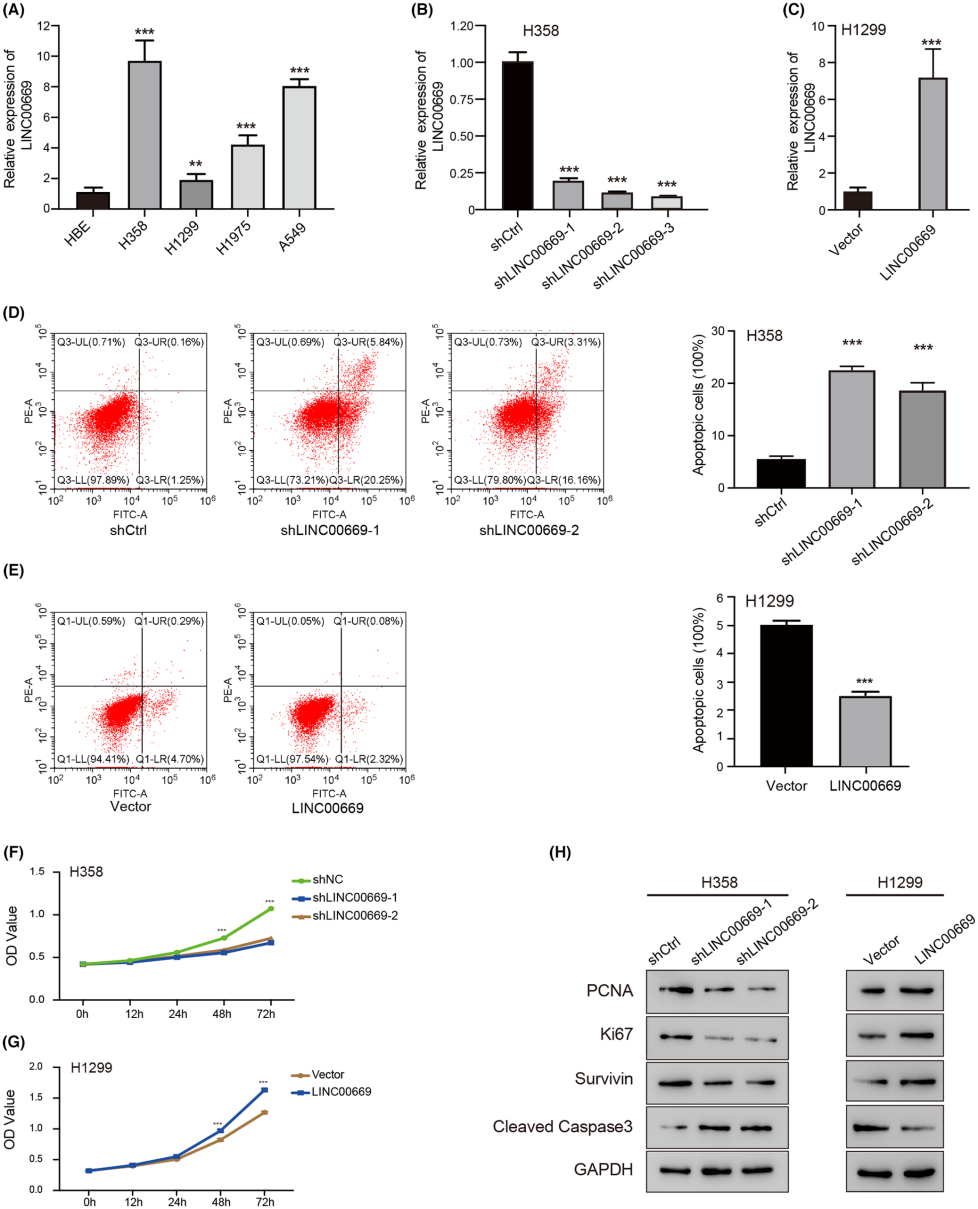
*LINC00669* promoted proliferation and suppressed apoptosis of NSCLC cells. (A) Basal expression levels of *LINC00669* in normal human lung bronchial epithelial cells (HEB) and four NSCLC cell lines as detected by qRT‐PCR. (B, C) *LINC00669* was successfully knocked down in H358 (B) and overexpressed NCI‐H1299 cells (C). (D, E) A flow cytometer was used to examine the effects of the depletion (D) or overexpression (E) of *LINC00669* on the apoptosis of NSCLC cells. (F, G) CCK‐8 assay shows proliferation at 24, 48, and 72 h for NSCLC cells with stable knockdown (F) or overexpression (G) of *LINC00669*. (H) Western blot results indicated the expression of PCNA, Ki67, surviving, and cleaved Caspase 3 in H358 (left) and 1299 (right) cells. The experiment was performed in triplicate, and data were calculated from three independent experiments using statistical analysis. *p < 0.05, **p < 0.01, ***p < 0.001.

Consistent with upregulated *LINC00669* in lung cancer, its expression levels were enhanced significantly in the indicated NSCLC cell lines than in normal HBE (human bronchial epithelial cells) cells (Figure 6A). *LINC00669* was knocked down in H358 and overexpressed in H1299 cells to investigate its role in lung cancer. After G418 (a neomycin analog) selection, *LINC00669* expression was lower in H358 cells with stable sh*LINC00669* transduction and higher in H1299 cells with stable expression of *LINC00669* than in corresponding control cells (Figure 6B,C). Flow cytometry results indicated that depletion of *LINC00669* induced apoptosis and reduced proliferation (Figure 6D,F). Opposite effects of *LINC00669* overexpression were also confirmed (Figure 6 E,H). Western blot analysis further verified that targeted delivery of sh *LINC00669* inhibited proliferation but promoted apoptosis of lung cancer cells. We detected decreased proliferation (PCNA and Ki67) and survival (survivin) makers, as well as increased apoptosis maker (cleaved‐Caspase 3) in *LINC00669*‐depleted H358 cells. Again, the forced expression of *LINC00669* showed reverse effects in H1299 cells (Figure 6H). These data showed a critical role of *LINC00669* in NSCLC cell proliferation and apoptosis.

### 3.8 Regulation of *LINC00669* on NSCLC cells is dependent on the Wnt signaling pathway

**FIGURE 7:**
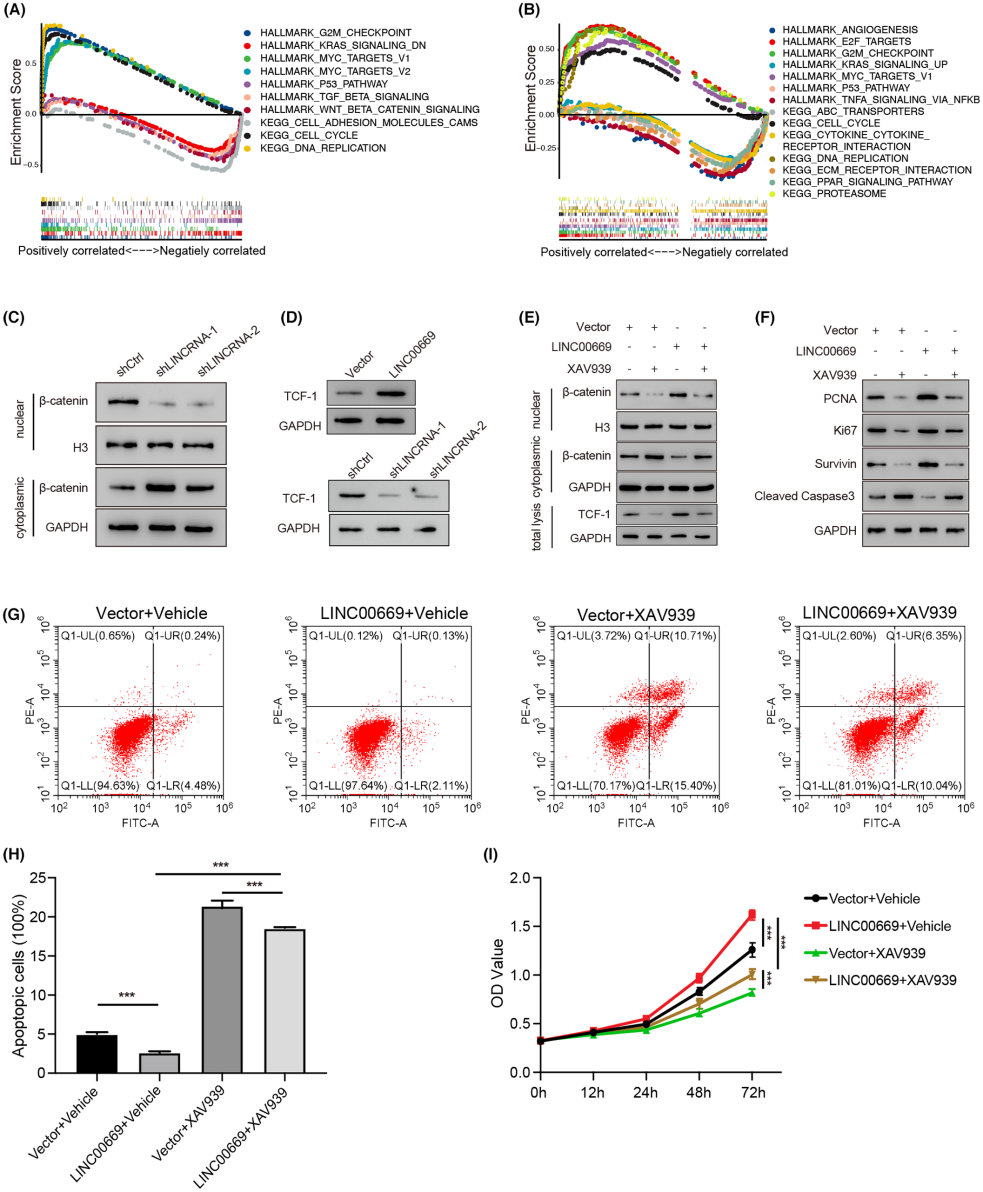
*LINC00669* controlled NSCLC cell proliferation and apoptosis by the Wnt signaling pathway. (A, B) GSEA revealed a number of signaling pathways associated with *LINC00669* in LUAD using the TCGA (A) and CCLE (B) databases. (C, D) Western blot results showed that the depletion or overexpression of *LINC00669* altered subcellular localization of β‐catenin and the expression levels of TCF‐1. (E) XAV939, the Wnt pathway inhibitor, reversed *LINC00669‐*induced activation of the Wnt signaling. (F) The levels of the proteins of interest in control and experiment cells in the presence or absence of XAV939. (G, H) Cell apoptosis was determined by flow cytometry and quantified. (I) CCK8 assay measured cell proliferation. The experiments were performed in triplicate, and data were calculated from three independent experiments using statistical analysis. *p < 0.05, **p < 0.01, ***p < 0.001.

We next explored the downstream signaling pathways by which *LINC00669* regulated lung cancer cell behaviors. GSEA results derived from TCGA‐LUAD (Figure 7A) and CCLE (Figure 7B) databases indicated that many signaling pathways were correlated with *LINC00669*. Using Western blot analysis, we validated that silencing *LINC00669* prevented β‐catenin from nuclear translocation and decreased downstream transcription factor T cell factor 1 (TCF‐1),^31^ suggesting inactivation of the Wnt signaling pathway (Figure 7C,D). We further performed a rescue experiment with XAV939, a Wnt pathway inhibitor. Western blot results exhibited that XAV939 and *LINC00669* overexpression inhibited and activated the Wnt pathway, respectively. XAV939 treatment could attenuate *LINC00669*‐induced nuclear translocation of β‐catenin and TCF‐1 upregulation (Figure 7E). Moreover, XAV939 treatment reversed *LINC00669*‐induced alteration in PCNA, Ki67, survivin, and cleaved Caspase 3 (Figure 7F). Flow cytometry analysis revealed that XAV939 ablated the inhibitory effects of *LINC00669* on lung cancer apoptosis (Figure 7G,H). CCK8 assay also verified that XAV939 could abolish *LINC00669*‐stimulated NSCLC cell proliferation (Figure 7I). These results provide evidence that *LINC00669* regulates lung cancer cells by activating the Wnt signaling pathway.

### 3.9 Validation of *LINC00669*'s oncogenic potential in vivo

**FIGURE 8:**
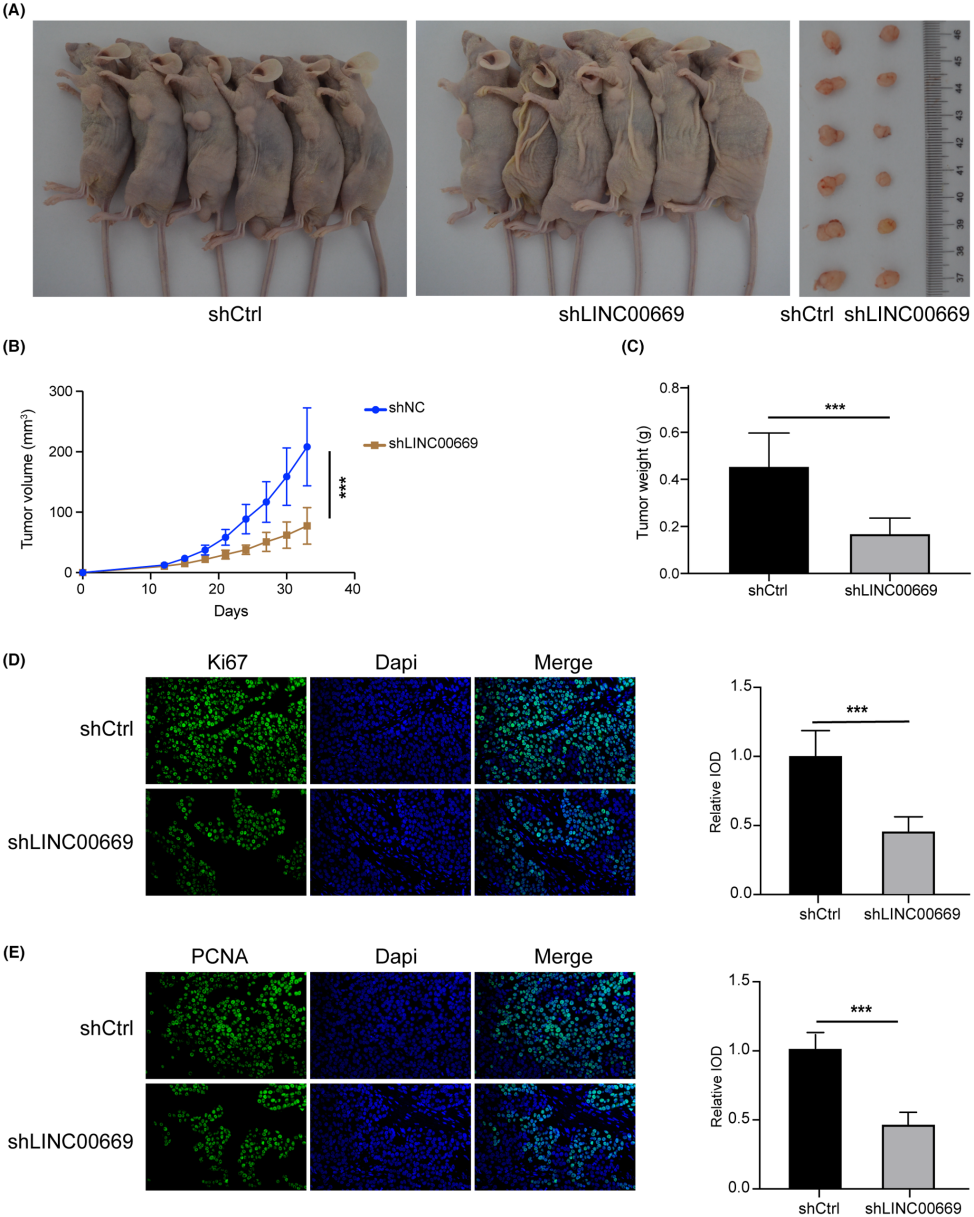
Verification of *LINC00669*'s oncogenic potentials in vivo. (A) Images of xenografts in nude mice, arising from H358 cells with stable knockdown of *LINC00669* and control cells. (B, C) Differences in tumor volumes (B) and tumor weights (C) between LV‐shCtrl and LV‐sh*INC00669* groups (*n* = 6). (D) Immunofluorescent staining of Ki67 on tumors for control and experimental groups (*n* = 6) and quantification of Ki67‐positive cells. (E) Immunofluorescent staining of PCNA on tumors for control and experimental groups and quantification of PCNA‐positive cells *p < 0.05, **p < 0.01, ***p < 0.001.

Given the regulation of *LINC00669* on lung cancer cells, we examined whether *LINC00669* depletion impedes NSCLC cells' tumor growth capacity in vivo. Control cells or cells stably infected with LV‐sh*LINC00669* were subcutaneously implanted into flanks of nude mice. Tumor volumes and weights were recorded. We found that LV‐sh*LINC00669* significantly shrank tumor size and reduced tumor weight when compared to the control (Figure 8A–C). IF staining indicated that the depletion of *LINC00669* significantly downregulated proliferation markers, Ki67 and PCNA, in xenografts (Figure 8D–E). Our results verified that targeting *LINC00669* restrains the carcinogenesis of NSCLC cells in vivo.

## 4 DISCUSSION

Lung cancer is a severe health problem worldwide, 85% of which is NSCLC. Over the past two decades, the treatment of NSCLC has been dramatically advanced by the development of specific targeted therapies and immune checkpoint inhibitors (ICIs).

However, the overall survival rates for NSCLC remain unsatisfying, especially in advanced diseases. Therefore, research is called for continuously improving the understanding of lung cancer biology and mechanisms of tumor progression, and discovering more novel therapeutic targets. Besides coding RNA, genomes are now well‐known to be transcribed and generate thousands of lncRNA, which have essential roles in gene regulation. Their regulatory mechanisms are closely linked to subcellular localization, involving the modulation of chromatin conformation, the assembly and function of membrane‐less nuclear bodies, the half‐life time and translation of cytoplasmic mRNAs, and the activity of signaling pathways.^32^ LncRNAs are frequently dysregulated in diverse physiopathological conditions, such as cancer.^7^

This study identified *LINC00669* as a novel oncogenic gene in NSCLC. The signature derived from *LINC00669*‐related cell cycle genes could effectively predict prognosis, TIME, and antitumor drug sensitivity. Moreover, in vitro and in vivo analyses demonstrated that *LINC00669* boosted NSCLC growth by arousing the Wnt signaling pathway.

Our GSEA unravels a tight linkage between *LINC00669* and the cell cycle, suggesting it may regulate lung cancer cell growth. Its potential cell cycle regulation inspired us to construct a predicting signature by integrating *LINC00669* and cell cycle genes. Traditionally, single genes are used to predict prognosis and drug sensitivity in cancer. However, cancer is a complex disease, which is hardly recapitulated by any single molecule. The identified single biomarkers are helpful but fail to fully represent lung cancer's complex mechanisms. Fortunately, striking advances in high‐throughput mRNA profiling techniques have made RNA‐seq affordable today. Over the past years, the machine learning approach coupled with RNA‐seq data has generated diverse gene signatures to improve the prediction of prognosis or therapeutic benefit.^33^, ^34^, ^35^, ^36^, ^37^, ^38^, ^39^, ^40^, ^41^ For instance, Li et al. built a powerful prognostic signature based on immune‐related gene pairs in early‐stage nonsquamous NSCLC.^35^ Yao et al. reported that a ferroptosis‐related lncRNA signature could predict prognosis in NSCLC.^41^

It was not surprising that *LINC00669* alone only has weak prognostic power with an AUC of 0.547, which might be attributed to the failure to consider its functional relevance to lung cancer development. Instead, virtual information concerning gene regulation on cancer cell survival could better portray candidate biomarkers' molecular implications in cancer progression and improve their predicting accuracy.^42^ Interestingly, *LINC00669*‐related cell cycle genes achieved an AUC of 0.700. While combined with risk score and stage, the AUC of *LINC00669* added up to 0.746. A growing body of evidence has shown success in establishing robust prognostic signatures by combining single molecular biomarkers and their biological relevance.^17^, ^23^, ^42^, ^43^ For instance, our group demonstrated that UBE2T stimulated autophagy in lung cancer cells in a preceding study. Based on this finding, we developed a signature of UBE2T‐related autophagy genes, which outperformed the single UBE2T gene in predicting the prognosis of LUAD.^23^ These results show that integrating biological implications is an effective strategy to enhance the predictive power of individual molecular biomarkers.

When we tried to dissect the differential molecular mechanism underneath the low‐and high‐risk group, GSVA disclosed high activity of some pathways in the high‐risk group, which are known to facilitate tumor cell survival and growth, including hypoxia, DNA repair, glycolysis, EMT, MYC targets V1, MYC targets V2, MTORC1 signaling, DNA replication, and regulation of autophagy. These dysregulated malignancy‐related molecular characteristics partially explain the inferior clinical outcomes in the high‐risk group.

Furthermore, our results showed that the signature of *LINC00669*‐related cell cycle genes was associated with the different TIMEs. It is well recognized that T cells (e.g., *T*_H_1 and cytotoxic T cells) play a central part in antitumor battle by releasing interferon‐γ (IFNγ), granulysin (GNLY), perforin (PRF1) and granzymes (GZMs), as wells as other mechanisms.^44^ By analyzing data retrieved from approximately 300 studies, Galon et al. found that CD8+ cytotoxic T cells had the most potent impacts on the prognosis of cancer patients among most tumor immune infiltrates.^26^ Intratumoral infiltrating CD8^+^ cytotoxic T cells, CD3^+^ cells, or CD45RO^+^ memory T cells have been reported to associate with favorable survival in various cancer.^26^, ^27^, ^45^ Hu et al. demonstrated that a high density of tumor‐infiltrating CD45RO+ memory cells was associated with prolonged survival in a Chinese LUAD patient population.^46^ CD4^+^ *T*_H_1 cells also participate in antitumor response by synthesizing transcription factor T‐bet and secreting IFNγ.^44^ In contrast, T regulatory cells are immune suppressive. In resected NSCLC, tumor‐infiltrating Foxp3+ regulatory T cells were associated with disease recurrence.^47^ We compared memory, cytotoxic, and immunosuppressive cells between the two groups by adopting an algorithm containing metagenes of 28 immune cells. The TIMEs in the two groups were significantly different regarding many subtypes of intratumoral immune cells. By analyzing the expression of ICPs, we further confirmed the different tumor immunogenicity between the two groups.

Immunophenoscore is a comprehensive determinant of tumor immunogenicity, calculated by integrating four critical types of tumor immunogenicity determinants: antigen processing (MHC), immune checkpoints and immunomodulators, effector cells (activated CD8, activated CD4, Tem CD4, Tem CD8, and suppressor cells [Treg and MDSC]). IPS has been validated to predict response to inhibitors CTLA and PD1. Consistently, our analysis indicated that the low‐risk groups have higher IPS than the high group, suggesting that the tumors in the low‐risk group are more immunogenic. Moreover, we also found that the low‐risk group has a tendency to respond to inhibitors of CTLA4. Our results align with others.^48^, ^49^ Yi et al. elucidated that the low risk of LUAD stratified by an immune‐related gene signature was highly likely to benefit from anti‐PD‐1/PDL‐1 and anti‐CTLA4 therapies.^48^ Besides, Mei and colleagues used a 4‐gene signature to define a low‐risk group of cervical cancer patients. Similarly, using IPS as a predictor of ICI response, these researchers found that the low‐risk group was more likely to benefit from ICIs than the high‐risk group.^49^ Our findings are in agreement with the concept that the “hot” immune tumors in the low‐risk groups are more likely to be inhibited by immunotherapy.^44^ These results unveiled that the current signature could also predict response to immunotherapy in LUAD.

We also validate the oncogenic role of *LINC00669* in NSCLC with in vitro and in vivo evidence. Qing et al. reported that *LINC00669* was enhanced in NPC and related to a poor prognosis.^18^ *LINC00669* triggered the JAK/STAT signaling pathway to stimulate NPC cell proliferation and invasion.^18^ The suppressor of cytokine signaling 1 (SOCS1) can mediate the ubiquitination of STAT1 and facilitate its degradation. *LINC00669* was found to decoy SOCS1 to prevent it from interacting with STAT1, thereby stabilizing STAT1 and activating the JAK/STAT signaling pathway.^18^ The implications of *LINC00669* in other tumors have not been reported, and its functions remain to be clarified. The present study indicated that *LINC00669* accelerates NSCLC growth by activating the Wnt signaling pathway.

The Wnt signaling tightly regulates lung development, regeneration, and disease progression, including lung cancer.^50^ SOX30 inhibited the Wnt signaling by directly repressing CTNNB1 transcription and competitively inhibiting the interaction of β‐catenin with TCF. SOX30 deficiency causes tumor metastasis in lung cancer by inducing Wnt signaling overactivation.^20^ In addition, Glypican‐5 (GPC5), downregulated in lung adenocarcinoma, could reduce tumor growth by suppressing Wnt/β‐catenin signaling.^19^ Guan et al. uncovered that a FOXM1 Variant (rs3742076_G) led to gefitinib resistance by enhancing the Wnt/β‐catenin activity.^21^ Overall, these results indicate that the Wnt/β‐catenin mediates the stimulatory impacts of *LINC00669* on the proliferation and apoptosis resistance of lung cancer cells.

In conclusion, *LINC00669* is a key oncogenic lncRNA in lung cancer. The signature of the *LINC00669*‐related cell cycle gene could distinguish two groups of LUAD patients with different survival, TIME, and anticancer drug sensitivity. Our results endorse the potential application of *LINC00669* as a biological marker and therapeutic target in lung cancer.

## AUTHOR CONTRIBUTIONS

**Jinhong Zhu:** Conceptualization (equal); data curation (equal); funding acquisition (equal); investigation (equal); methodology (equal); project administration (equal); supervision (equal); writing – original draft (lead). **Kui Cao:** Data curation (equal); methodology (equal); visualization (equal). **Ping Zhang:** Investigation (supporting); validation (supporting). **Jianqun Ma:** Conceptualization (supporting); funding acquisition (lead); project administration (lead); writing – review and editing (lead).

## FUNDING INFORMATION

This study received financial support from the Natural Science Foundation of China (82172786), the Natural Science Foundation of Heilongjiang Province (LH2021H077), and the National Cancer Center Climbing Fund of China (NCC201908B06).

## CONFLICT OF INTEREST

The authors declare no conflict of interest.

## ETHICAL APPROVAL

The protocol of animal studies received approval from the Institutional Review Board of Harbin Medical University Cancer Hospital.

## Supporting information


Figure S1.

Table S1.

## Data Availability

The data generated in this study may be accessible upon reasonable request. Public datasets utilized here were obtained from the following websites: the TCGA database (https://cancergenome.nih.gov/), GEO database (https://www.ncbi.nlm.nih.gov/geo/), the CCLE database (https://portals.broadinstitute.org/ccle/), the CancerSEA database (http://biocc.hrbmu.edu.cn/CancerSEA/), Molecular Signatures Database v7.1 (MSigDB, https://www.gsea‐msigdb. org/gsea/index.jsp), and the TCIA database (https://tcia.at/home).
