# Supplementary figures and images for: LINC00669 promotes lung adenocarcinoma growth by stimulating the Wnt/β‐catenin signaling pathway
Source: Cancer Med. 2023 Jan 9;12(7):9005–23. doi: 10.1002/cam4.5604 (PMC10134358; doi:10.1002/cam4.5604)

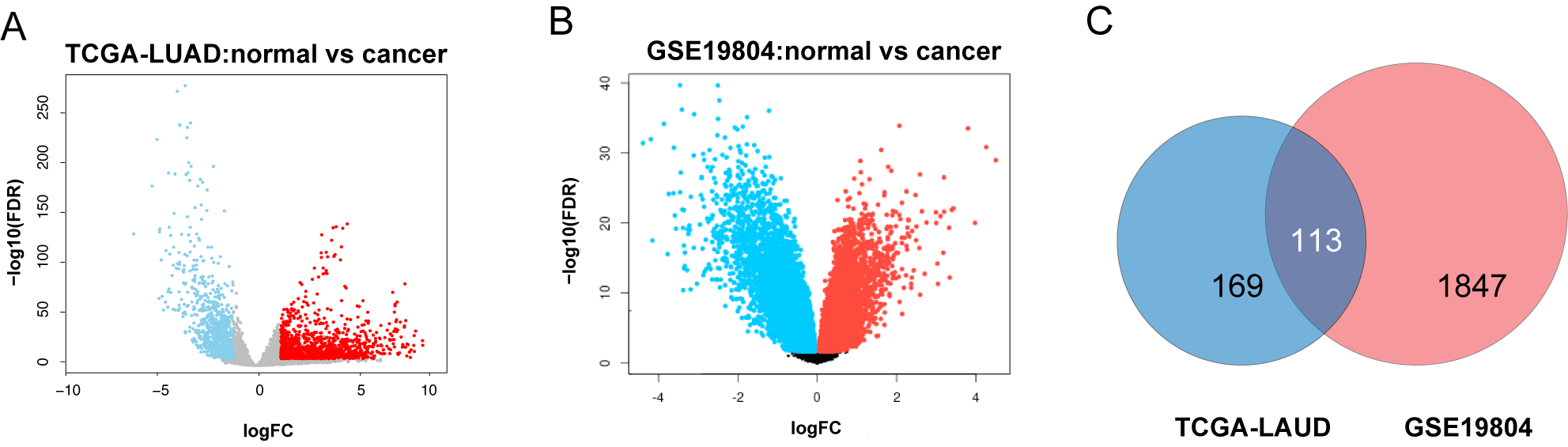

Supplement: Supplementary file 1 — Figure S1. [file CAM4-12-9005-s001.tif]
