# Supplementary material for: LINC00669 promotes lung adenocarcinoma growth by stimulating the Wnt/β‐catenin signaling pathway
Source: Cancer Med. 2023 Jan 9;12(7):9005–23. doi: 10.1002/cam4.5604 (PMC10134358; doi:10.1002/cam4.5604)
Supplement: Supplementary file 2 — Table S1. [file CAM4-12-9005-s002.docx]

| Supplemental Table 1. Differentially expressed and prognostic lncRNAs in LUAD. | | | | | |
| --- | --- | --- | --- | --- | --- |
| Gene ID | log2FC | *P* ^a^ | HR | 95% CI | *P* ^b^ |
| GAS1RR | -1.623743438 | 4.45E-19 | 0.564 | 0.419-0.758 | 0.000149984 |
| LMO7DN | -1.544888227 | 5.07E-13 | 0.566 | 0.422-0.759 | 0.000146053 |
| C2orf91 | -2.899786936 | 3.35E-32 | 0.583 | 0.435-0.782 | 0.00031131 |
| DRAIC | 3.811509647 | 2.46E-30 | 0.615 | 0.460-0.823 | 0.001045584 |
| FAM30A | 1.590015988 | 2.17E-12 | 0.625 | 0.467-0.837 | 0.00161597 |
| LINC01238 | 1.637812431 | 1.80E-14 | 0.661 | 0.494-0.884 | 0.005309346 |
| FENDRR | -3.933385788 | 1.19E-75 | 0.693 | 0.519-0.925 | 0.01277012 |
| LINC00115 | 1.521083027 | 8.93E-29 | 0.705 | 0.526-0.945 | 0.019192935 |
| WASIR2 | 3.481003284 | 6.18E-47 | 0.719 | 0.539-0.959 | 0.024587314 |
| LRRK2-DT | -1.981236359 | 3.92E-13 | 0.739 | 0.553-0.987 | 0.040568858 |
| LINC00968 | -3.562477966 | 1.01E-74 | 0.745 | 0.559-0.993 | 0.044853931 |
| MIR924HG | 3.826272672 | 5.96E-47 | 1.802 | 1.345-2.416 | 8.04E-05 |
| HR, hazard ratio; CI, confidence interval; FC, Fold Change. MIR924HG is another name for LINC00669.  ^a^ T-test for differences in lncRNA expression between tumors and normal tissues.  ^b^ Univariate Cox regression analysis for lncRNAs in the TCGA-LUAD cohorts. | | | | | |
